# Supplementary material for: Enhanced Levels of Glycosphingolipid GM3 Delay the Progression of Diabetic Nephropathy
Source: Int J Mol Sci. 2023 Jul 12;24(14):11355. doi: 10.3390/ijms241411355 (PMC10379152; doi:10.3390/ijms241411355)
Supplement: Supplementary file 1 [file ijms-24-11355-s001.zip › ijms-2472725-supplementary.pdf]

**Suppplementary Table S1** Reagents, Antibodies used in this study

| Name | Source | Type |
|---|---|---|
| Streptozotocin | FUJIFILM Wako, #195-15154 | powder |
| Valproic acid sodium salt | FUJIFILM Wako, #193-18352 | powder |
| anti-Insulin | Invitrogen, #14-9769-82 | Mouse, monoclonal |
| anti-p57 (H-91) | Santa Cruz Biotech, #sc-8298 | Rabbit, polyclonal |
| anti-PDGFR-β (28E1) | Cell Signaling Tech, #3169 | Rabbit, monoclonal |
| anti-SGLT2 | abcam, #ab85626 | Rabbit, polyclonal |
| anti-Nephrin C-terminus | Original [1] | Mouse, polyclonal |
| Anti-GM3 (GMR6) | Tokyo chemical Industry, #A2582 | Mouse, monoclonal |
| Alexa Fluor 488-Rabbit anti-Mouse IgG | Thermo Fisher Scientific, #A27023 | Rabbit, polyclonal |
| Alexa Fluor 568-Goat anti-Mouse IgM | Life Technologies, #A-21043 | Goat, polyclonal |

**Reference**

1. Kawashima, N.; Naito, S.; Hanamatsu, H.; Nagane, M.; Takeuchi, Y.; Furukawa, J. I.; Iwasaki, N.; Yamashita, T.; Nakayama, K. I., Glycosphingolipid GM3 prevents albuminuria and podocytopathy induced by anti-nephrin antibody. *Sci Rep* **2022,** 12, (1), 16058. DOI: 10.1038/s41598-022-20265-w
